# Statistical analysis plan for a cluster randomised trial in Madhya Pradesh, India: support to rural India’s public education system and impact on numeracy and literacy scores (STRIPES2)

**DOI:** 10.1186/s13063-023-07453-3

**Published:** 2023-07-22

**Authors:** Suzanne Keddie, Ila Fazzio, Siddharudha Shivalli, Nicholas Magill, Diana Elbourne, Dropti Sharma, Sajjan Singh Shekhawat, Rukmini Banerji, Sridevi Karnati, Harshavardhan Reddy, Alex Eble, Peter Boone, Chris Frost

**Affiliations:** 1 London School of Hygiene and Tropical Medicine, London, UK; 2 Effective Intervention, London, UK; 3 Pratham Education Foundation, New Delhi, India; 4 GH Training and Consulting, Hyderabad, India; 5 Teachers College, Columbia University, New York, USA

## Abstract

**Background:**

India has made steady progress in improving rates of primary school enrolment but levels of learning achievement remain low. The Support To Rural India’s Public Education System (STRIPES) trial provided evidence that an after-school para-teacher intervention improved numeracy and literacy levels in Telangana, India. The STRIPES2 trial investigates whether such an intervention will have a similar effect on the literacy and numeracy of primary school age children in the Satna District of Madhya Pradesh, India.

**Methods/design:**

The STRIPES2 trial forms one part of a cluster-randomised controlled trial with villages (clusters) randomised to receive either a health (CHAMPION2) or education (STRIPES2) intervention. Building on the design of the earlier CHAMPION/STRIPES trial, villages receiving the health intervention are controls for the education intervention and vice versa. The primary outcome is a combined literacy and numeracy score. Secondary outcomes include separate scores for literacy and numeracy; caregivers’ engagement with child’s learning; expenditure on education; enrolment in school; caregiver’s report of school attendance and the cost effectiveness of the intervention. Over 7000 primary school age children have been recruited and randomised in STRIPES2.

**Discussion:**

This update to the published trial protocol gives a detailed plan for the statistical analysis of the STRIPES 2 trial.

**Trial registration:**

Registry of India: CTRI/2019/05/019296. Registered on 23 May 2019. http://www.ctri.nic.in/Clinicaltrials/pdf_generate.php?trialid=31198&EncHid=&modid=&compid=%27,%2731198det%27

## Introduction

### Background and rationale

India has made steady progress in improving rates of primary school enrolment. In rural areas, about 97% of children between 6 and 14 years of age are now in school [1]. The levels of learning achievement, however, remain low. The 2018 Annual Status of Education Report (ASER) survey showed that proficiency in reading and numeracy is worryingly low and Indian children may spend several years in school without learning even the basic skills in literacy and numeracy [1]. The STRIPES trial and subsequent SCORE trial intervention demonstrated important results in improving numeracy and language scores in Telangana, India [2] and rural Gambia [3]. The STRIPES 2 trial [4] investigates whether such an intervention will have a similar effect on the literacy and numeracy of primary school age children in Satna District of Madhya Pradesh, India.

### Objectives

The primary objective is to assess whether the success of the STRIPES and SCORE trials in providing an after-school para-teacher intervention to raise learning levels among primary school students in rural India and rural Gambia can be replicated in Satna district of Madhya Pradesh, India.

The primary outcome is a combined literacy and numeracy score. Secondary outcomes include separate scores for literacy and numeracy; caregivers’ engagement with child’s learning; expenditure on education; enrolment in school; caregiver’s report of school attendance and the cost effectiveness of the intervention.

## Study methods

### Trial design

This is a cluster-randomised controlled trial where the recruited clusters are villages in the Satna district of Madhya Pradesh, India. The villages included satisfied the following criteria:

1. Were considered rural, with fewer than 2500 population and with more than 120 children under the age of 6 years;
2. Were accessible by road;
3. Weren’t within a 5 km radius of the Community Health Centres (as such villages are already well-served by the local health services);
4. Had a minimum of 3 km between village centres, such buffer zones being included to minimize contamination.

From a baseline survey conducted between July 2017 and January 2018 we enrolled children born between 16 June 2010 and 15 June 2013 whose caregivers were planning to enrol them in the first grade, for the first time, in the 2018–2019 school year in eligible villages. Before randomization of villages, from April-June 2019, we conducted a catch-up enumeration in all the selected villages to enrol eligible children who were missed during the baseline enumeration (this included some children who were by this time attending school). Villages were allocated in a 1:1 ratio to either the intervention (a programme provided by Pratham intending to provide remedial out-of-school lessons, focusing on literacy and numeracy, 6 days a week, 2 h a day for 17 months), or to control.

Planned daily classes were temporarily stopped in compliance with government measures to reduce COVID-19 transmission from April-Dec 2020 and May–June 2021. The intervention was restarted with modifications according to the local COVID-19 guidelines such as daily small group and weekly (for children who couldn’t attend daily classes) classes. The intervention period was also extended by 12 months, ending in June 2022.

Between 24^th^ July and 19^th^ September 2022 participant children in both trial arms were tested with Early Grade Reading Assessment (EGRA) [5] and the Early Grade Mathematics Assessment (EGMA) [6] tests adapted to the local language and context. After the testing all the children were given a small set of school material as recompense for their time.

### Randomisation

Randomisation of clusters was performed by the trial statistician based in London in June 2019 using a random number generator, with stratification by village size and distance to the nearest Community Health Centre or Civil Hospital.

### Sample size

The relevant parts of the original sample size calculation as published in the protocol were as follows.

Originally it had been the intention to randomise 300 villages, because this gave over 90% statistical power to detect a difference of 0.25 standard deviations in mean standardised test scores in STRIPES 2. However, incorporating the buffer zones described in the village selection procedure above meant that only 204 villages could be selected. These 204 villages have a mean population of 1487 (minimum 558, maximum 2490) and a standard deviation of 505 (equating to a coefficient of variation of 0.34). Estimating the number of children in each school year from the number under the age of six years old (divided by 6), the mean number of children in each school year is 38.3 (minimum 20, maximum 71) with a standard deviation of 13.3 (a coefficient of variation of 0.35). Assuming that 25% of the children will not satisfy the eligibility criteria, this gives an estimated mean number of eligible children per village of 28.7 with a minimum of 15.

We estimated that the 204 villages will include an average of 28.7 eligible students. In the STRIPES trial the estimated effect was a 0.75 SD increase in mean score: however, effects of smaller magnitude than this would still be important to detect. Conservatively assuming that 60% of the eligible children will take the test at the end of the trial, and an intra-cluster correlation coefficient of 0.23 (as seen in the STRIPES trial [2]), then a trial with 194 villages (i.e. assuming that 5% of the 204 villages will not take part) will give 88% power to detect a difference of 0.25 SD in mean standardised scores between intervention and control villages using a conventional 2-sided statistical significance level of 5% (assuming a coefficient of variation in numbers taking the test by village of 0.35). If the treatment effect is of the order of that seen in the STRIPES trial then there will be reasonable statistical power to explore interactions by ethnicity, gender, wealth and geographic location.

As described above, in the sample size calculation we anticipated that 194 of the 204 villages would be randomised. In fact, 196 were randomised, as 6 villages were removed since they were found to be too close to urban areas to be considered rural, and 2 removed because insufficient eligible children were found. Over 7000 children were enumerated in the randomised villages, with over 6000 children taking the test at the end of follow-up.

### Framework

The trial will use a superiority hypothesis testing framework.

### Statistical interim analyses and stopping guidance

As no potential harms are anticipated from this intervention, there is no Data Monitoring Committee, interim analyses or stopping rules.

### Timing of final analysis

May 2023 to August 2023.

### Timing of outcome assessments

The primary outcome (the endline composite mathematics and language score) was assessed through endline tests (EGRA and EGMA) carried out between 24^th^ July and 19^th^ September 2022.

Additional data collection was carried out as follows:

- Between January and February 2022, a midline test was carried out with the children to assess basic reading and mathematics levels using an ASER-like exam.
- Between February and April 2022, a midline survey was carried out with the caregivers to record enrolment, reported attendance and educational support during the period that schools were closed.
- In November and December 2022, a final survey was carried out to record changes in school enrolment and reported attendance, and caregivers’ support to child’s education.
- Throughout the trial, data on attendance in classes in the intervention arm were collected by Pratham.

## Statistical principles

### Level of statistical significance

5%

### Adjustments for multiplicity

None (not applicable).

### Confidence intervals to be reported

Yes, 95% confidence intervals.

### Definition of adherence to the intervention and how this is assessed including extent of exposure

Villages did not all run the intervention classes in the same way. There was variability in the number of planned classes per week, the length of these and the size of classes. Also, some children who lived far from classes in their village could not be reached. This was further complicated by COVID-19 when schools were closed and no after-school classes were running. This makes calculation of measures of adherence challenging. For simplicity we will simply use counts of the numbers of classes i) offered to and ii) attended by each child. We also assume that, had the intervention run as planned, then each child would have been offered 360 classes (6 classes a week for 60 weeks, this corresponding approximately to a 17-month period with allowance for holidays etc.). We refer to this as the ideal number of classes.

For the *j*th child in the *i*th village we will calculate, over the full follow-up period i) the total number of classes that were offered to that child ($${O}_{ij}$$) and ii) the total number of classes that that child attended ($${A}_{ij}).$$

At child level we will define adherence in three ways.

- Attended as a proportion of ideal (${A}_{ij}/360$).
- Offered as a proportion of ideal $({O}_{ij}/360)$.
- Attended as a proportion of offered (${A}_{ij}/{O}_{ij}$).

At village level, using ${N}_{i}$ to denote the number of children in the* i*th village, we will define adherence in the same three ways.

- Attended as a proportion of ideal $\left({\sum }_{j}{A}_{ij}\right)/\left(360{N}_{i}\right)$.
- Offered as a proportion of ideal $\left({\sum }_{j}{O}_{ij}\right)/\left(360{N}_{i}\right)$.
- Attended as a proportion of offered $\left({\sum }_{j}{A}_{ij}\right)/\left({\sum }_{j}{O}_{ij}\right)$.

Each measure will be summarised using means and standard deviations, and in a contingency table with adherence bands of (0, > 0 to 25%, > 25% to 50%, > 50% to 75%, > 75% to 100%, Table 1).

**Table 1:** Adherence, intervention arm only

	Adherence measure					
	Child level measures			Cluster level measures		
	Attended as a proportion of ideal (*N* = 3419)	Offered as a proportion of ideal (*N* = 3419)	Attended as a proportion of offered (*N* = 3419)	Attended as a proportion of ideal (*N* = 98)	Offered as a proportion of ideal (*N* = 98)	Attended as a proportion of offered (*N* = 98)
mean (SD)	x (x)	x (x)	x (x)	x (x)	x (x)	x (x)
0	n (%)	n (%)	n (%)	n (%)	n (%)	n (%)
> 0 to 25%	n (%)	n (%)	n (%)	n (%)	n (%)	n (%)
> 25% to 50%	n (%)	n (%)	n (%)	n (%)	n (%)	n (%)
> 50% to 75%	n (%)	n (%)	n (%)	n (%)	n (%)	n (%)
> 75% to 100%	n (%)	n (%)	n (%)	n (%)	n (%)	n (%)
Missing	n (%)	n (%)	n (%)	n (%)	n (%)	n (%)

### Definition of protocol deviations for the trial

Deviation from the protocol is defined as either 1) an intervention village not receiving any of the intervention during the trial intervention period, or 2) a control village receiving the intervention during the trial intervention period. Such protocol deviations will be listed.

### Analysis populations

The primary analysis will follow the intention to treat principle.

For the primary outcome two secondary per-protocol analyses will be performed, one corresponding to each of the “attended as a proportion of ideal” measures of adherence defined above. In each case the per-protocol analysis will be restricted to those with adherence at above 75%.

## Trial population

### Screening data

**Fig. 1:**
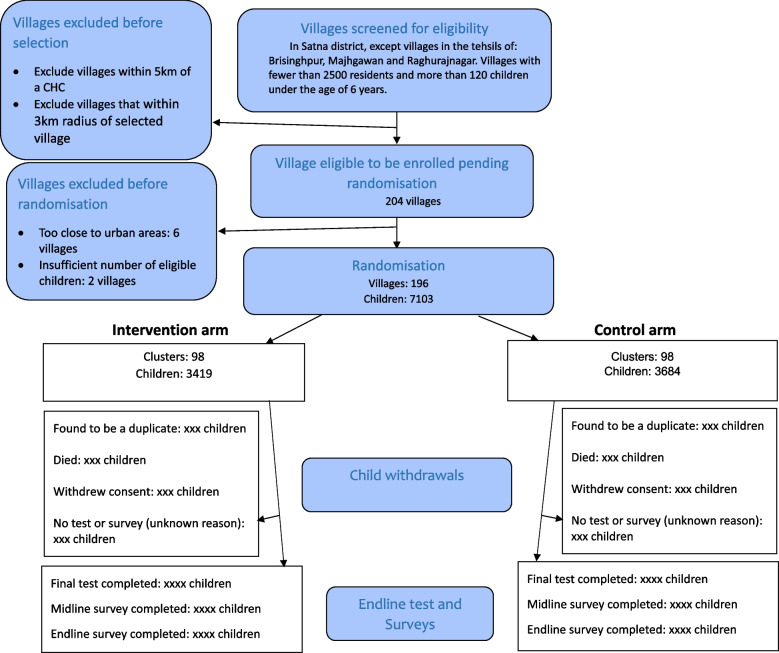
CONSORT flow diagram

The CONSORT Flow diagram summarises the identification, randomisation and reasons for withdrawal of villages and children within the trial. The diagram (shown in Fig. 1) will show numbers of villages approached but not randomised, with reasons listed.

### Eligibility criteria

A village was potentially eligible if the following conditions were met:

1. Village in Satna district, except villages in the tehsils of: Birsinghpur, Majhgawan and Raghurajnagar;
2. Village population less than 2500;
3. Village has more than 120 children under the age of 6 and at least 15 children eligible for the intervention;
4. Village is accessible by road;
5. Village centre is at least 5 km from a Community Health Centre (CHC);
6. Village centre is at least 3 km from the centre of any other included village.

A child was eligible if he or she was resident in a village within an eligible cluster at the time of enumeration, and fit the following criteria:

1. He or she did not attend first grade or higher in the 2017 – 2018 academic year;
2. He or she was expected to be resident in the village during 2018 – 2019:
3. The child’s caregiver intended to enrol the child in the first grade in the 2018 – 2019 academic year;
4. He or she was born between 16 June 2010 and 15 June 2013;
5. The caregiver consented to allow the child to participate in the trial.

A child was also eligible during the catch-up enumeration (carried out before randomisation) if:

1. He or she was born between 16 June 2010 and 15 June 2013;
2. He or she was enrolled in first grade in the 2018 – 2019 academic year or was planning to enter first grade in the 2019 – 2020 academic year;
3. He or she was expected to be resident in the village during 2019 – 2020;
4. The caregiver consented to allow the child to participate in the trial.

### Recruitment Information to be included in the CONSORT flow diagram

This is described in the Trial Population section.

### Withdrawal/follow-up

No clusters withdrew from the trial.

Children who have withdrawn will be considered to be those enrolled children whose caregivers subsequently rescinded consent for the child’s participation in the trial.

Loss to follow-up for the primary outcome will be considered to be children who do not attend both endline tests. For secondary outcomes, loss to follow-up will be considered to be children whose caregiver was not interviewed at the endline survey.

### Baseline patient characteristics

The following baseline characteristics will be tabulated by treatment arm. No baseline hypothesis tests will be carried out. For categorical variables the overall proportions (with numerators and denominators) will be shown as will the mean and standard deviation of the cluster level proportions. For continuous variables the overall mean and standard deviation will be shown along with the mean and standard deviation of the cluster level means.

Cluster-level variables (Table 2):

- Village size
- Distance to community health center/civic hospital

**Table 2:** Baseline characteristics of villages

Variable	Intervention arm *N* = 98	Control arm *N* = 98
Village size (total population)		
Mean (SD)	x (x)	x (x)
Median (IQR)	x (x)	x (x)
Distance (km) to nearest Community Hospital/Community Health Centre		
Mean (SD)	x (x)	x (x)
Median (IQR)	x (x)	x (x)

Individual-level variables (Table 3):

- Gender
- Child’s age
- Religion
- Caste
- Primary female caregiver (*i.e.*, mother or other)
- Literacy of female primary caregiver
- Education level of female primary caregiver
- Primary male caregiver (*i.e.*, father or other)
- Literacy of male primary caregiver
- Education level of male primary caregiver
- Parents still alive at baseline
- Wealth index 1. Determined by the material the house is made of: 1. Floor, roof and wall materials all natural, 2. Some, but not all, of floor, roof and wall materials are synthetic, 3. Floor, roof and wall materials all synthetic (as in Eble et al., 2020) [3].
- Wealth index 2. Number of Items (television, radio, motorbike, 4-wheeled vehicle) owned by the household members.

**Table 3:** Baseline characteristics

Variable	Intervention arm		Control arm	
	Individual level *N* = 3419	Cluster level *N* = 98 mean (SD)	Individual level *N* = 3684	Cluster level *N* = 98 mean (SD)
Proportion female	n (%)	x (x)	n (%)	x (x)
Family Religion:				
Hindu	n (%)	x (x)	n (%)	x (x)
Muslim	n (%)	x (x)	n (%)	x (x)
Family Caste:				
Schedule Caste	n (%)	x (x)	n (%)	x (x)
Schedule Tribe	n (%)	x (x)	n (%)	x (x)
Other Backward Caste	n (%)	x (x)	n (%)	x (x)
Forward Caste	n (%)	x (x)	n (%)	x (x)
Missing	n (%)	x (x)	n (%)	x (x)
Child’s main female caregiver				
Biological mother	n (%)	x (x)	n (%)	x (x)
Step mother	n (%)	x (x)	n (%)	x (x)
Grandmother	n (%)	x (x)	n (%)	x (x)
Other female family member	n (%)	x (x)	n (%)	x (x)
Other	n (%)	x (x)	n (%)	x (x)
No female caregiver	n (%)	x (x)	n (%)	x (x)
Missing	n (%)	x (x)	n (%)	x (x)
Child’s main male caregiver				
Biological father	n (%)	x (x)	n (%)	x (x)
Step father	n (%)	x (x)	n (%)	x (x)
Grandfather	n (%)	x (x)	n (%)	x (x)
Other male family member	n (%)	x (x)	n (%)	x (x)
Other	n (%)	x (x)	n (%)	x (x)
No male caregiver	n (%)	x (x)	n (%)	x (x)
Missing	n (%)	x (x)	n (%)	x (x)
Main female caregiver's education:				
No schooling	n (%)	x (x)	n (%)	x (x)
Primary	n (%)	x (x)	n (%)	x (x)
Middle School	n( %)	x (x)	n( %)	x (x)
High School	n (%)	x (x)	n (%)	x (x)
Higher secondary	n (%)	x (x)	n (%)	x (x)
Graduate	n (%)	x (x)	n (%)	x (x)
Postgraduate	n (%)	x (x)	n (%)	x (x)
Missing	n (%)	x (x)	n (%)	x (x)
Main male caregiver's education:				
No schooling	n (%)	x (x)	n (%)	x (x)
Primary	n (%)	x (x)	n (%)	x (x)
Middle School	n (%)	x (x)	n (%)	x (x)
High School	n (%)	x (x)	n (%)	x (x)
Higher secondary	n (%)	x (x)	n (%)	x (x)
Graduate	n (%)	x (x)	n (%)	x (x)
Postgraduate	n (%)	x (x)	n (%)	x (x)
Missing	n (%)	x (x)	n (%)	x (x)
Child’s age	mean (SD)	x (x)	mean (SD)	x (x)
Mother alive at baseline	n (%)	x (x)	n/N	x (x)
Father alive at baseline	n (%)	x (x)	n/N	x (x)
Main female caregiver's literacy:				
Can’t read	n (%)	n (%)	n (%)	n (%)
Can read part of the sentence	n (%)	n (%)	n (%)	n (%)
Read entire sentence	n (%)	n (%)	n (%)	n (%)
Missing	n (%)	n (%)	n (%)	n (%)
Main male caregiver's literacy:				
Can’t read	n (%)	n (%)	n (%)	n (%)
Can read part of the sentence	n (%)	n (%)	n (%)	n (%)
Read entire sentence	n (%)	n (%)	n (%)	n (%)
Missing	n (%)	n (%)	n (%)	n (%)

## Analysis

### Outcomes

The primary outcome of the trial is the composite literacy and numeracy test score using the EGRA and EGMA, respectively (Table 4 with subgroup analysis in Table 5). A sensitivity analysis will be carried out omitting the score from EGRA subtask 5b question 1, which was judged to be potentially misleading.

**Table 4:** EGRA and EGMA test results

Variable	Intervention arm		Control arm		Difference (95% CI) *p*-value
	Individual level N: mean (SD)	Cluster level *N* = 98 mean (SD)	Individual level N: mean (SD)	Cluster level *N* = 98 mean (SD)	
Composite test score	N: x (x)	x (x)	N: x (x)	x (x)	x (x, x) *p* = x
Composite test score - sensitivity analysis	N: x (x)	x (x)	N: x (x)	x (x)	x (x, x) *p* = x
Mathematics test score, overall	N: x (x)	x (x)	N: x (x)	x (x)	x (x, x) *p* = x
Mathematics test, combined fluency scores	N: x (x)	x (x)	N: x (x)	x (x)	
Mathematics test, combined untimed subtasks	N: x (x)	x (x)	N: x (x)	x (x)	
Mathematics 1	N: x (x)	x (x)	N: x (x)	x (x)	
Mathematics 2	N: x (x)	x (x)	N: x (x)	x (x)	
Mathematics 3	N: x (x)	x (x)	N: x (x)	x (x)	
Mathematics 4a	N: x (x)	x (x)	N: x (x)	x (x)	
Mathematics 4b	N: x (x)	x (x)	N: x (x)	x (x)	
Mathematics 5a	N: x (x)	x (x)	N: x (x)	x (x)	
Mathematics 5b	N: x (x)	x (x)	N: x (x)	x (x)	
Mathematics 6	N: x (x)	x (x)	N: x (x)	x (x)	
Language test score, overall	N: x (x)	x (x)	N: x (x)	x (x)	x (x, x) *p* = x
Language test score, overall - sensitivity analysis	N: x (x)	x (x)	N: x (x)	x (x)	x (x, x) *p* = x
Language test, combined fluency scores	N: x (x)	x (x)	N: x (x)	x (x)	
Language test, combined untimed subtasks	N: x (x)	x (x)	N: x (x)	x (x)	
Language 1	N: x (x)	x (x)	N: x (x)	x (x)	
Language 2	N: x (x)	x (x)	N: x (x)	x (x)	
Language 3	N: x (x)	x (x)	N: x (x)	x (x)	
Language 4	N: x (x)	x (x)	N: x (x)	x (x)	
Language 5a	N: x (x)	x (x)	N: x (x)	x (x)	
Language 5b	N: x (x)	x (x)	N: x (x)	x (x)	
Language 5b - sensitivity analysis	N: x (x)	x (x)	N: x (x)	x (x)	
Language 6	N: x (x)	x (x)	N: x (x)	x (x)

**Table 5:** Composite test scores by subgroup, with interaction tests

Subgroup	Intervention arm		Control arm		Difference (95% CI)	*P*-value
	Individual level N: mean (SD)	Cluster level N: mean (SD)	Individual level N: mean (SD)	Cluster level N: mean (SD)		
Village population						
Below median	N: x (x)	N: x (x)	N: x (x)	N: x (x)	x (x, x)	*p* = x
Above median	N: x (x)	N: x (x)	N: x (x)	N: x (x)	x (x, x)	
Gender						
Male	N: x (x)	N: x (x)	N: x (x)	N: x (x)	x (x, x)	*p* = x
Female	N: x (x)	N: x (x)	N: x (x)	N: x (x)	x (x, x)	
Wealth Index 1						
Category 1	N: x (x)	N: x (x)	N: x (x)	N: x (x)	x (x, x)	*p* = x
Category 2	N: x (x)	N: x (x)	N: x (x)	N: x (x)	x (x, x)	
Category 3	N: x (x)	N: x (x)	N: x (x)	N: x (x)	x (x, x)	
Wealth Index 2 (items owned)						
0	N: x (x)	N: x (x)	N: x (x)	N: x (x)	x (x, x)	
1	N: x (x)	N: x (x)	N: x (x)	N: x (x)	x (x, x)	*p* = x (trend test)
2	N: x (x)	N: x (x)	N: x (x)	N: x (x)	x (x, x)	
3	N: x (x)	N: x (x)	N: x (x)	N: x (x)	x (x, x)	
4	N: x (x)	N: x (x)	N: x (x)	N: x (x)	x (x, x)	
Caste						
Schedule Caste	N: x (x)	N: x (x)	N: x (x)	N: x (x)	x (x, x)	*p* = x
Schedule Tribe	N: x (x)	N: x (x)	N: x (x)	N: x (x)	x (x, x)	
Other Backward Caste	N: x (x)	N: x (x)	N: x (x)	N: x (x)	x (x, x)	
Forward Caste	N: x (x)	N: x (x)	N: x (x)	N: x (x)	x (x, x)	
Female Caregiver Literacy						
Can’t read	N: x (x)	N: x (x)	N: x (x)	N: x (x)	x (x, x)	*p* = x
Can read part of the sentence	N: x (x)	N: x (x)	N: x (x)	N: x (x)	x (x, x)	
Read entire sentence	N: x (x)	N: x (x)	N: x (x)	N: x (x)	x (x, x)	
Male Caregiver Literacy						
Can’t read	N: x (x)	N: x (x)	N: x (x)	N: x (x)	x (x, x)	*p = x*
Can read part of the sentence	N: x (x)	N: x (x)	N: x (x)	N: x (x)	x (x, x)	
Read entire sentence	N: x (x)	N: x (x)	N: x (x)	N: x (x)	x (x, x)	

Secondary outcomes include the separate scores for literacy and numeracy; caregivers’ engagement on child learning; enrolment in school at the end of follow-up; caregiver’s report of school attendance and the cost effectiveness of the intervention.

Secondary outcomes to be formally tested and a 95% confidence interval constructed are as follows.

- Mathematics test score, to be calculated as a simple arithmetic mean of the percentage of correct answers on each of the six (some composite) subtasks, evenly weighting each task and not accounting for time remaining. The six subtasks are 1, 2, 3, 4 [mean of 4a and 4b], 5 [mean of 5a and 5b] and 6 (Table 4).
- Language test score, to be calculated as a simple arithmetic mean of the percentage of correct answers on each of the seven subtasks, evenly weighting each task and not accounting for time remaining. The seven subtasks are 1, 2, 3, 4, 5a, 5b and 6. A sensitivity analysis will be carried out omitting the score from EGRA subtask 5b question 1, which was judged to be potentially misleading (Table 4).
- Midline test scores (mathematics and language, Table 6).
- Whether child is enrolled in school at the endline survey (Table 7).
- Number of hours caregiver spends engaging child in reading or writing activities post lockdown (Table 8).
- Caregiver’s report of school attendance; number of days of school missed in the past two weeks, conditional on enrollment. As recorded in the endline survey (Table 9).
- Cost per 0.1 standard deviation improvement in the primary outcome. The standard deviation to be estimated by fitting a linear mixed model with cluster-specific random effects to the primary outcome in the control arm of the trial, with the standard deviation estimated via a summation of the between- and within-cluster variances. The included costs will be all costs for running the intervention and any capital costs will be amortized according to the item. It will include all costs that would occur if the trial intervention were continued without the research costs related to a trial. It does not reflect the costs that a government organization would observe if they took over the intervention. It does not include any costs to families.

**Table 6:** Midline test results

Variable	Intervention arm		Control arm		Difference (95% CI) *p*-value
	Individual level N: mean (SD)	Cluster level *N* = 98 mean (SD)	Individual level N: mean (SD)	Cluster level *N* = 98 mean (SD)	
Mathematics test score	N: x (x)	x (x)	N: x (x)	x (x)	x (x, x) *p* = x
Beginner level	n (%)	n (%)	n (%)	n (%)	
Numbers 1–9	n (%)	n (%)	n (%)	n (%)	
Numbers 10–99	n (%)	n (%)	n (%)	n (%)	
Addition	n (%)	n (%)	n (%)	n (%)	
Subtraction	n (%)	n (%)	n (%)	n (%)	
Language test score	N: x (x)	x (x)	N: x (x)	x (x)	x (x, x) *p* = x
Beginner level	n (%)	n (%)	n (%)	n (%)	
Letters	n (%)	n (%)	n (%)	n (%)	
Words	n (%)	n (%)	n (%)	n (%)	
Paragraph	n (%)	n (%)	n (%)	n (%)	
Story	n (%)	n (%)	n (%)	n (%)

**Table 7:** Children enrolled in school

Variable	Intervention arm		Control arm		Odds ratio^a^ (95% CI) *p*-value
	Individual level *N* = 3419	Cluster level *N* = 98 mean (SD)	Individual level *N* = 3684	Cluster level *N* = 98 mean (SD)	
Midline – pre lockdown					
Yes	n (%)	x (x)	n (%)	x (x)	
No	n (%)	x (x)	n (%)	x (x)	
Missing	n (%)	x (x)	n (%)	x (x)	
Midline – post lockdown					
Yes	n (%)	x (x)	n (%)	x (x)	
No	n (%)	x (x)	n (%)	x (x)	
Missing	n (%)	x (x)	n (%)	x (x)	
Endline					
Yes	n (%)	x (x)	n (%)	x (x)	x (x, x) *p* = x
No	n (%)	x (x)	n (%)	x (x)	
Missing	n (%)	x (x)	n (%)	x (x)	

^a^Yes v No ignoring missing

**Table 8:** Learning support (endline)

Variable	Intervention arm		Control arm		Difference (95% CI) *p*-value
	Individual level *N* = 3419	Cluster level *N* = 98 mean (SD)	Individual level *N* = 3684	Cluster level *N* = 98 mean (SD)	
Help for home study					
No	n (%)	x (x)	n (%)	x (x)	
Yes	n (%)	x (x)	n (%)	x (x)	
Missing	n (%)	x (x)	n (%)	x (x)	
Hours (ignoring missing)	mean (SD)	mean (SD)	mean (SD)	mean (SD)	x (x, x) *p* = x

**Table 9:** Reported attendance in school, among those enrolled (endline)

	**Intervention arm**		**Control arm**		Difference (95% CI) *p*-value
	Individual level *N* = xxxx	Cluster level *N* = 98 mean (SD)	Individual level *N* = xxxx	Cluster level *N* = 98 mean (SD)	
Number of days of school missed in the last two weeks					
0	n (%)	x (x)	n (%)	x (x)	
1	n (%)	x (x)	n (%)	x (x)	
2	n (%)	x (x)	n (%)	x (x)	
3	n (%)	x (x)	n (%)	x (x)	
4	n (%)	x (x)	n (%)	x (x)	
…	n (%)	x (x)	n (%)	x (x)	
Missing	n (%)	x (x)	n (%)	x (x)	
Mean (SD)	x (x)	x (x)	x (x)	x (x)	x (x, x) *p* = x

Secondary outcomes to be tabulated but not formally tested

- Mathematics test score on the combined timed subtasks, to be calculated as a simple arithmetic mean of the fluency measures on each of timed subtasks (Table 4).
- Language test score on the combined timed subtasks, to be calculated as a simple arithmetic mean of the fluency measures on each of the timed subtasks (Table 4).
- Mathematics test score on the combined untimed subtasks, to be calculated as a simple arithmetic mean of the percentage of correct answers on each of the subtasks, evenly weighting each task (Table 4).
- Language test score on the combined untimed subtasks, to be calculated as a simple arithmetic mean of the percentage of correct answers on each of the subtasks, evenly weighting each task (Table 4).
- Whether child is enrolled in school pre- and post the covid lockdown (midline survey, Table 7).
- Child’s residence status (Table 10).
  - ◦ Data sources:
    - ▪ Midline
    - ▪ Endline
- Grade (number 0–5) child is enrolled in during each phase of the trial (Table 11).
  - ◦ Data sources:
    - ▪ Midline pre lockdown:
    - ▪ Midline post lockdown:
    - ▪ Endline:

- Challenges faced during COVID-19 lockdown (Table 12).
  - ◦ Any challenges faced?
  - ◦ Specific challenges faced:
    - ▪ No smartphone
    - ▪ Limited access to smartphone
    - ▪ Internet connectivity issues
    - ▪ Internet costs too expensive
    - ▪ Electricity Issues
    - ▪ Lack of school teacher support
    - ▪ Lack of time to help child
    - ▪ Low knowledge of technology
    - ▪ Child not interested
    - ▪ No money for a private tutor

**Table 10:** Children resident in study village

Variable	Intervention arm		Control arm	
	Individual level N = 3419	Cluster level N = 98 mean (SD)	Individual level N = 3684	Cluster level N = 98 mean (SD)
Midline				
Yes	n (%)	x (x)	n (%)	x (x)
No	n (%)	x (x)	n (%)	x (x)
Missing	n (%)	x (x)	n (%)	x (x)
Endline				
Yes	n (%)	x (x)	n (%)	x (x)
No	n (%)	x (x)	n (%)	x (x)
Missing	n (%)	x (x)	n (%)	x (x)

**Table 11:** School grade of child

Variable	Intervention arm		Control arm	
	Individual level *N* = 3419	Cluster level *N* = 98 mean (SD)	Individual level *N* = 3684	Cluster level *N* = 98 mean (SD)
Midline – pre lockdown				
Anganwadi	n (%)	x (x)	n (%)	x (x)
Pre-primary	n (%)	x (x)	n (%)	x (x)
1	n (%)	x (x)	n (%)	x (x)
2	n (%)	x (x)	n (%)	x (x)
3	n (%)	x (x)	n (%)	x (x)
4	n (%)	x (x)	n (%)	x (x)
..	n (%)	x (x)	n (%)	x (x)
Don’t know	n (%)	x (x)	n (%)	x (x)
Missing	n (%)	x (x)	n (%)	x (x)
Midline – post lockdown				
Anganwadi	n (%)	x (x)	n (%)	x (x)
Pre-primary	n (%)	x (x)	n (%)	x (x)
1	n (%)	x (x)	n (%)	x (x)
2	n (%)	x (x)	n (%)	x (x)
3	n (%)	x (x)	n (%)	x (x)
4	n (%)	x (x)	n (%)	x (x)
..	n (%)	x (x)	n (%)	x (x)
Don’t know	n (%)	x (x)	n (%)	x (x)
Missing	n (%)	x (x)	n (%)	x (x)
Endline				
Anganwadi	n (%)	x (x)	n (%)	x (x)
Pre-primary	n (%)	x (x)	n (%)	x (x)
1	n (%)	x (x)	n (%)	x (x)
2	n (%)	x (x)	n (%)	x (x)
3	n (%)	x (x)	n (%)	x (x)
4	n (%)	x (x)	n (%)	x (x)
..	n (%)	x (x)	n (%)	x (x)
Don’t know	n (%)	x (x)	n (%)	x (x)
Missing	n (%)	x (x)	n (%)	x (x)

**Table 12:** Covid-19 challenges faced (midline)

Variable	Intervention arm		Control arm	
	Individual level *N* = 3419 mean (SD)	Cluster level *N* = 98 mean (SD)	Individual level *N* = 3684 mean (SD)	Cluster level *N* = 98 mean (SD)
Any challenges faced?				
No	n (%)	x (x)	n (%)	x (x)
Yes	n (%)	x (x)	n (%)	x (x)
Missing	n (%)	x (x)	n (%)	x (x)
Specific challenges				
No smartphone	n (%)	x (x)	n (%)	x (x)
Limited access to smartphone	n (%)	x (x)	n (%)	x (x)
Internet connectivity issues	n (%)	x (x)	n (%)	x (x)
Internet costs too expensive	n (%)	x (x)	n (%)	x (x)
Electricity issues	n (%)	x (x)	n (%)	x (x)
Lack of schoolteacher support	n (%)	x (x)	n (%)	x (x)
Lack of time to help child	n (%)	x (x)	n (%)	x (x)
Low knowledge of technology	n (%)	x (x)	n (%)	x (x)
Child not interested	n (%)	x (x)	n (%)	x (x)
No money for a private tutor	n (%)	x (x)	n (%)	x (x)

- Learning support provided by family, school teachers, NGOs and/or private tutors during the time when schools were closed (Table 13).
  - ◦ Help at home to study
  - ◦ Educational activities using online videos, recorded classes or games found on educational mobile learning apps/websites
  - ◦ Educational activities using textbooks or worksheets
  - ◦ Source of textbooks/worksheets (schoolteacher, caregiver/family, NGOs, private tutor.
  - ◦ Purchased items by family to specifically support education:
    - ▪ Smart phone
    - ▪ Tablet
      ▪ Computer

**Table 13:** Learning support (midline)

Variable	Intervention arm		Control arm	
	Individual level *N* = 3419 mean (SD)	Cluster level *N* = 98 mean (SD)	Individual level *N* = 3684 mean (SD)	Cluster level *N* = 98 mean (SD)
Help for home study				
No	n (%)	x (x)	n (%)	x (x)
Yes	n (%)	x (x)	n (%)	x (x)
Don’t know	n (%)	x (x)	n (%)	x (x)
Missing	n (%)	x (x)	n (%)	x (x)
Home devices				
Regular phone bought	n (%)	x (x)	n (%)	x (x)
Smartphone bought	n (%)	x (x)	n (%)	x (x)
Tablet/computer bought	n (%)	x (x)	n (%)	x (x)
Access at home to				
Regular phone	n (%)	x (x)	n (%)	x (x)
Smartphone Tablet/computer	n (%)	x (x)	n (%)	x (x)
Educational videos etc	n (%)	x (x)	n (%)	x (x)
Textbooks or worksheets	n (%)	x (x)	n (%)	x (x)
Support from schools				
Learning materials/activities	n (%)	x (x)	n (%)	x (x)
Child’s progress/well-being	n (%)	x (x)	n (%)	x (x)
Administrative information	n (%)	x (x)	n (%)	x (x)

- Spending on school materials, school fees and out of school tuition (Table 14)

**Table 14:** Spending (midline) in Rupees

	**Intervention arm**		**Control arm**	
	Individual level *N* = 3419 mean (SD)	Cluster level *N* = 98 mean (SD)	Individual level *N* = 3684 mean (SD)	Cluster level *N* = 98 mean (SD)
School materials	x (x)	x (x)	x (x)	x (x)
School fees	x (x)	x (x)	x (x)	x (x)
Out of school tuition	x (x)	x (x)	x (x)	x (x)
Other	x (x)	x (x)	x (x)	x (x)

### Analysis methods

In the primary analysis of the primary outcome, child-specific composite test scores at endline will be compared between intervention and control arms using a linear regression model with randomisation arm and the stratification factors (and no other variables) as predictor variables. To take account of the cluster-randomisation, robust standard errors, allowing for the clustering, will be used here and elsewhere. Linear mixed models (with cluster as a random effect) which are also termed hierarchical or multilevel models are commonly used for the analysis of cluster randomised trials. The advantage of an approach using robust standard errors over linear mixed models is that homoscedasticity assumptions are not made.

The adjusted difference in means will be divided by the SD of the test score in the control arm to give a standardised difference, with a nonparametric bootstrap confidence interval (bias corrected and accelerated, 2000 replications at cluster level) computed for this.

Secondary outcomes that are continuous will be analysed using the same approach as above.

Secondary analyses will extend the linear regression model (with robust standard errors that allow for clustering) for the primary outcome described above to (separately) investigate interactions by caste, gender, male and female primary caregiver literacy, village population and wealth.

Secondary outcomes that are dichotomous (such as whether the child was enrolled in school) will be expressed as odds ratios with 95% confidence intervals obtained from a GEE model with a binary outcome, a logit link, and a ‘working’ assumption of independence, with robust standard errors to take account of clustering.

### Adjustment for covariates

These are described in the Analysis methods section above.

### Methods used for assumptions to be checked for statistical methods

The linear regression models used for the primary analysis assume that residuals are normally distributed. Robust standard errors allow for potential heteroscedasticity according to levels of predictor variables, but do make an assumption of normality conditional on levels of predictor variables. This assumption will be checked by examination of appropriate quantile–quantile plots of standardised residuals. The central limit theorem ensures that results are robust provided that violations of the normality assumptions are not substantial. Minor violations, even if statistically significant, are of little practical consequence. For this reason, formal hypothesis tests of normality assumptions will not be carried out.

### Alternative methods to be used if distributional assumptions do not hold

Nonparametric bootstrap confidence intervals (bias corrected and accelerated, 2000 replications at cluster level) will be reported if the normality assumptions are seriously violated.

### Sensitivity analyses for each outcome where applicable

In the primary analysis, missing data will not be imputed. In secondary analyses of the primary outcome and key secondary outcomes, multiple imputation by chained equations (MICE) will be used. For analysis of clustered data it is important that the model for imputation includes cluster-specific random effects [7]. Such analyses will be carried out using the Jumo package within the statistical package R [8]. Imputation will be carried out separately in each trial arm. Auxiliary variables to potentially be used will include the randomisation stratification factors, caste, gender, male and female primary caregiver literacy, the wealth indices, the adherence to intervention variables defined above, the midline test scores, enrolment at endline, the number of hours the caregiver spends engaging child in reading or writing activities post lockdown, the caregiver’s report of school attendance, whether or not the child is enrolled in school pre- and post the covid lockdown, school grade at endline, the child’s residence status and the variables quantifying the learning support (and spending) provided by family, school teachers, NGOs and/or private tutors during the time when schools were closed.

If the effect of the intervention is statistically significant, and remains so in the MICE analysis detailed above then the multiple imputation analysis will also be extended to determine the amount of bias over and above that allowed for by the multiple imputation model that would render the primary analysis non- statistically significant.

### Subgroup analyses

We will conduct subgroup analyses (Table 5) of the primary outcome by.

- Gender
- Wealth index 1 (in three categories determined by the material the house is made of)
- Wealth index 2 (in five categories determined by the number of relevant items owned by the household, with the interaction tested using a trend test).
- Caste
- Primary female caregiver literacy in 3 groups. This to be replaced by female education if more than 10% of the participants have a missing value for literacy and education status is not missing.
- Primary male caregiver literacy in 3 groups. This to be replaced by male education if more than 10% of the participants have a missing value for literacy and education status is not missing.
- Village population (above/below median)

For each of the above factors, statistical tests for interaction will be carried out, with claims of different effects in subgroups only made if there is strong evidence (*p* < 0.01) of an interaction.

### Reporting and assumptions/statistical methods to handle missing data (e.g., multiple imputation)

These are described in the Sensitivity analysis section above.

### Additional analyses

Additional analysis to be conducted include an economic evaluation calculating total average cost, and total average cost per 0.1 standard deviation improvement in the primary outcome. The standard deviation to be estimated by fitting a linear mixed model with cluster-specific random effects to the primary outcome in the control arm of the trial, with the standard deviation estimated via a summation of the between- and within-cluster variances. The included costs will be all costs for running the intervention and any capital costs will be amortized according to the item. It will include all costs that would occur if the trial intervention were continued without the research costs related to a trial. It does not reflect the costs that a government organization would observe if they took over the intervention. It does not include any costs to families.

Also, as a result of the COVID-19 lockdowns, additional support was provided to enrolled children and their mothers. Summary data relating to this will be tabulated. Data collected included the number of direct messages sent to children and the response rate to these messages, the number of home-visits received, attendance of mothers in fortnightly meetings to encourage engagement, access to and use of books at local libraries and, access to and use of a tablet providing digital learning.

### Statistical software

Stata version 17 (StataCorp. 2021. Stata Statistical Software: Release 17. College Station, TX: StataCorp LLC) and/or R (R Core Team 2022. R: A language and environment statistical computing. R Foundation for Statistical Computing, Vienna, Austria. URL https://www.R-project.org/).

## Trial status and declarations

### Trial status

The statistical analysis plan is based on the published protocol [4].

This is a cluster randomised trial, with all villages (clusters) randomised in 2019. Eligible children for the STRIPES2 trial were all enrolled prior to randomisation. Endline tests and surveys for STRIPES2 were conducted in 2022. Data cleaning for STRIPES2 is ongoing with possible return to the field for outstanding queries, prior to anticipated data-lock in May 2023.

### Data management plan

The final EGRA and EGMA (literacy and numeracy) tests will be double-entered in the main office of the research team in Satna. The database has been developed by Sealed Envelope (https://www.sealedenvelope.com), an independent company contracted to construct and maintain a bespoke database for the trial, who will also keep a periodical backup of the data.

### Trial master file, statistical master file and standard operating procedures

The trial master file is part of the standard operating procedures manual. The standard operating procedures manual is available upon request. The statistical master file is held securely and may be available upon request after final analyses. 

## Data Availability

Data sharing is not applicable to this article (a statistical analysis plan) as no datasets will be generated or analysed during this stage of the study. After publication of the initial results, the anonymised datasets used and/or analysed during the trial with relevant statistical code will be available from the corresponding author on reasonable request.
